# Genome Sequence of Human Papillomavirus Type 20, Strain HPV-20/Lancaster/2015

**DOI:** 10.1128/genomeA.00712-17

**Published:** 2017-08-03

**Authors:** Kate V. Atkinson, Lisa A. Bishop, Glenn Rhodes, Nicolas Salez, Neil R. McEwan, Matthew J. Hegarty, Julie Robey, Nicola Harding, Simon Wetherell, Robert M. Lauder, Roger W. Pickup, Mark Wilkinson, Derek Gatherer

**Affiliations:** a Division of Biomedical & Life Sciences, Faculty of Health & Medicine, Lancaster University, Lancaster, United Kingdom; b Royal Lancaster Infirmary, Lancaster, United Kingdom; c Centre for Ecology & Hydrology, Lake Ecosystems Group, Lancaster Environment Centre, Lancaster University, Lancaster, United Kingdom; d UMR_D 190, Emergence des Pathologies Virales, Aix-Marseille University, Marseille, France; e Institute of Biological, Environmental & Rural Sciences, Aberystwyth University, Aberystwyth, United Kingdom; f Queen Square Medical Practice, Lancaster, United Kingdom

## Abstract

The genome sequence of human papillomavirus type 20 (HPV-20; family *Papillomaviridae*, genus *Betapapillomavirus*, species *Betapapillomavirus 1*, type 20) was assembled by deep sequencing from nasopharyngeal swabs. The assembled genome is 0.37% divergent over its full length from the single complete genome of HPV-20 in GenBank (U31778). We named the strain HPV-20/Lancaster/2015.

## GENOME ANNOUNCEMENT

The family *Papillomaviridae* consists of more than 120 viral types, divided into 49 genera (1). Within the genus *Betapapillomavirus*, type 20 of the species *Betapapillomavirus 1* (HPV-20) is represented in GenBank by a single complete genome (2), a circular double-stranded DNA of 7.8 kb (U31778), and three shorter fragments (3–5). HPV-20 is a clinically significant papillomavirus, having been implicated in epidermodysplasia verruciformis (2), skin cancer (6), ocular syringoma (7), nongenital seborrhoeic keratosis (8), conjunctival papillomas (9), verrucous carcinoma of the lip (10), condylomatous lesions of the mammillae (11), squamous cell carcinoma of the esophagus (12), and toenail onycholysis (13).

Volunteers were recruited from a general practice surgery and a general hospital in Lancaster, UK (54.05°N, 2.80°W). Nasopharyngeal swabs were taken between 16 December 2014 and 25 February 2015. Ethical approval was granted by the UK National Research Ethics Service, reference 14/LO/1634, NIHR Clinical Research Network (UKCRN) portfolio, ID 17799. All methods were carried out in accordance with the relevant guidelines and regulations.

Pooled RNA from 51 swabs was deep sequenced using an Illumina Nextera XT library and HiSeq 2500 system (SRA accession no. SRP092324). An HPV-20 genome was assembled using BWA 0.7.12-r1039 (14), with U31778 as the template. The assembled genome is 7,742 bases in length, differing from U31778 by 29 substitutions (0.37%). A 15-base deletion in the new genome starts at position 110, within an AT repeat that has a total length of 52 residues in U31778. Three regions cannot be resolved: (i) 3351 to 3353, (ii) 3704 to 3736; and (iii) 3798 to 3839. The second and third of these are in repetitive regions. We cannot exclude the possibility that the lengths of these repetitive regions differ between U31778 and the new genome, as we have shown for the region around the deletion at position 110. The predicted protein sequences are derived by reference to U31778 and differ at 14 amino acid residues (0.53%), without nonsense substitutions.

de Villiers et al. (15) recommend that a nucleotide divergence of 15% be used as the threshold for designation of a new type of human papillomavirus. The new strain is therefore well within the range of diversity expected within type 20 and has been designated HPV-20/Lancaster/2015. It is only the second full-length genome of HPV-20 to be described.

Forslund et al. (16) found HPV types of the *Betapapillomavirus 1* species in 17% of nasal swabs in a study of 312 Danish health care staff members, but HPV-20 was detected in only 6 individuals (1.9%). Of 9 deep-sequenced nonoverlapping subsets of individuals from our 51 swabs, we detected HPV-20 in all but 2. Our frequency is therefore at least 7/51 (>13%) and possibly much higher. The significance of this clinically important papillomavirus at such prevalence in our sample remains a matter for speculation.

BAM files are available from https://doi.org/10.17635/lancaster/researchdata/145. 

### Accession number(s).

The genome sequence of HPV-20/Lancaster/2015 has been deposited in GenBank under the accession number KY969593.
